# TORC2: a novel target for treating age-associated memory impairment

**DOI:** 10.1038/srep15193

**Published:** 2015-10-22

**Authors:** Jennifer L. Johnson, Wei Huang, Gregg Roman, Mauro Costa-Mattioli

**Affiliations:** 1 Department of Neuroscience, Baylor College of Medicine, Houston, TX 77030, USA; 2 Memory and Brain Research Center (MBRC), Baylor College of Medicine, Houston, TX 77030, USA; 3 Biology and Biochemistry Department, University of Houston, Houston, TX 77004, USA; 4 Biology of Behavior Institute, University of Houston, Houston, TX 77004, USA

## Abstract

Memory decline is one of the greatest health threats of the twenty-first century. Because of the widespread increase in life expectancy, 20 percent of the global population will be over 60 in 2050 and the problems caused by age-related memory loss will be dramatically aggravated. However, the molecular mechanisms underlying this inevitable process are not well understood. Here we show that the activity of the recently discovered mechanistic target of rapamycin (mTOR) complex 2 (mTORC2) declines with age in the brain of both fruit flies and rodents and that the loss of mTORC2-mediated actin polymerization contributes to age-associated memory loss. Intriguingly, treatment with a small molecule that activates mTORC2 (A-443654) reverses long-term memory (LTM) deficits in both aged mice and flies. In addition, we found that pharmacologically boosting either mTORC2 or actin polymerization enhances LTM. In contrast to the current approaches to enhance memory that have primarily targeted the regulation of gene expression (epigenetic, transcriptional, and translational), our data points to a novel, evolutionarily conserved mechanism for restoring memory that is dependent on structural plasticity. These insights into the molecular basis of age-related memory loss may hold promise for new treatments for cognitive disorders.

During the past century, the global population has witnessed a dramatic increase in life expectancy^1^. Because individuals are living much longer, cognitive decline has emerged as one of the greatest health threats of old age. Currently, no treatment is available to reverse or delay age-associated cognitive decline. Without an effective intervention, it is estimated that, by 2040, over 90 million people will experience age-mediated memory deficits^2^. Thus, the development of novel and efficient cognitive enhancers to treat these disorders is of crucial importance. However, in order to develop a therapy that can restore memory in the aging brain, we must first have a better understanding of the molecular and neuronal mechanisms underlying age-associated memory impairment.

Normal aging of the human brain is accompanied by robust and progressive alterations in cognition, mood and motor function^3^,^4^. A decline in these behaviors with age is highly correlated with structural and neurophysiological changes in the brain^5^. Previous studies have shown that the aging brain undergoes a non-uniform loss of grey and white matter volumes, including accelerated shrinkage of the hippocampus and entorhinal cortices^4^,^6^. Interestingly, these morphological changes are not a result of increased neuronal death, but rather due to shrinkage of neuronal dendritic arbors and loss of synapses^7^,^8^. The question remains whether dysregulated molecular signaling pathways in specific cognitive domains can elicit age-associated structural changes.

Although the evolutionarily conserved mechanistic target of rapamycin (mTOR) has been implicated in aging^9^, its role in brain aging remains unclear. mTOR forms two functionally distinct complexes. The first complex, mTORC1, consisting of mTOR, Raptor and mLST8 (GβL), is sensitive to the immunosuppressant rapamycin and regulates mRNA translation rates^10^,^11^. The second complex, mTORC2, which was recently discovered, is largely insensitive to rapamycin and consists of mTOR, mSIN1, mLST8 and Rictor^12^,^13^,^14^. Although much less is known about its down-stream effectors and up-stream regulation, mTORC2 has been shown to regulate the actin cytoskeleton and seems to play an important role in brain function^15^,^16^,^17^. Indeed, by regulating actin polymerization, mTORC2 controls the structural changes at synapses that are necessary for memory consolidation^15^. Furthermore, mTORC2 activity is altered in several age-associated cognitive disorders, including Alzheimer’s disease^18^ and Parkinson’s disease^19^, in which age is the major risk factor.

Current approaches to reverse memory loss and enhance memory in the aged population have focused on changes in gene expression at the epigenetic^20^,^21^ and transcriptional^22^ levels. However, changes in synaptic actin polymerization are also crucially involved in memory formation^23^,^24^,^25^. Given that a) human brain aging is associated with memory loss^3^,^4^,^5^, b) specific regions of the aging brain exhibit reduced synaptic connectivity^7^,^8^, c) inhibition of mTORC2 decreases life span^26^ and d) mTORC2 regulates structural changes required for memory consolidation^15^,^16^,^17^, we investigated the role of mTORC2 deficiency as a novel mechanism of age-associated memory loss, and the therapeutic potential of mTORC2 as a target for the treatment of memory loss in aged animals.

## Results

### TORC2 activity decreases with age in flies

**Figure 1:**
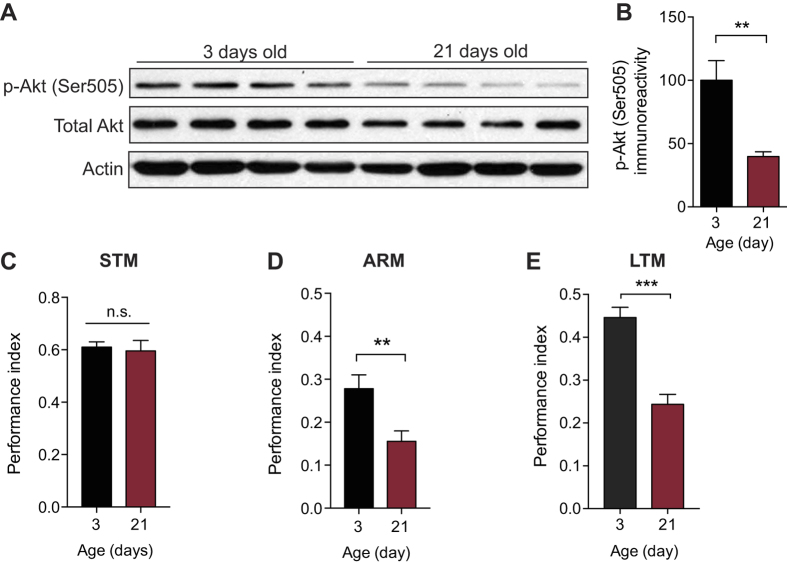
dTORC2 activity is reduced and long-lasting memory is impaired in aged flies. (**A**) Western blot analysis revealed a significant decrease in dTORC2 activity (p-Akt Ser505) in brain extracts from aged (21 d) WT flies compared to young (3 d) WT flies. Actin is the loading control and the blot is representative of three independent replicates. (**B**) Normalized data of p-Akt Ser505 levels (p-Akt Ser505 normalized to total Akt levels; n = 4 per group, unpaired *t* test, t = 3.771, **P < 0.01). (**C-E**) STM is unaffected in aged (21 d) WT flies (C; n = 6 per group, unpaired *t* test, t = 0.326, P = 0.751), but both forms of long-lasting memory, ARM (D; n = 10 per group, unpaired *t* test, t = 3.025, **P < 0.01) and protein synthesis-dependent LTM (E; n = 6 per group, unpaired *t* test, t = 6.102, ***P < 0.001), are significantly impaired. All data are presented as mean ± s.e.m.

TORC2 promotes LTM storage and long-lasting changes in synaptic function^15^. Given the successful use of *Drosophila* as a model of age-related disorders^27^,^28^,^29^, we first decided to utilize the fly to investigate the mechanisms that contribute to age-associated memory loss. We determined by Western blotting the levels of *Drosophila* TORC2 (dTORC2) activity in the brain of wild-type flies at different ages by measuring the phosphorylation of Akt at Ser505, an established readout of dTORC2 activity^30^. We found that, compared to the brain of young (3 day old) flies, the brain of aged (21 day old) flies contained significantly less phosphorylated Akt (Ser505) (Figs. 1A-B). Hence, dTORC2 activity is reduced in the brain of aged flies.

### Aged flies are selectively impaired in long-lasting memory

A decline in LTM is a consequence of aging that is shared between humans and flies^29^,^31^,^32^,^33^. Given that the mechanisms underlying LTM formation in flies are highly conserved across species^34^, we investigated the effect of age on different temporal phases of memory in flies. We therefore tested memory in young and aged flies using olfactory classical conditioning, a well-studied form of associative learning^35^. In this paradigm, flies were trained to associate an odor, the conditioned stimulus (CS), with an aversive electric shock, the unconditioned stimulus (US). This conditioned memory can be dissected into different phases: a short-term memory and two forms of long-lasting memory, anesthesia-resistant memory (ARM)—that lasts only a few days— and a more persistent long-term memory (LTM)^36^. ARM is formed after either a single associative learning trial or several consecutive trials (massed protocol) whereas LTM is formed only after multiple spaced training trials. Unlike ARM, LTM is protein synthesis - dependent^36^,^37^. We observed a significant impairment of both ARM and LTM (Figs. 1D-E) in aged flies, though their short-term memory (STM, measured 3 min post-training) was not affected (Fig. 1C). Consistent with previous findings^32^,^33^, aged flies have a selective impairment in long-lasting memories.

### Direct activation of dTORC2 and actin polymerization restores LTM in aged flies

**Figure 2:**
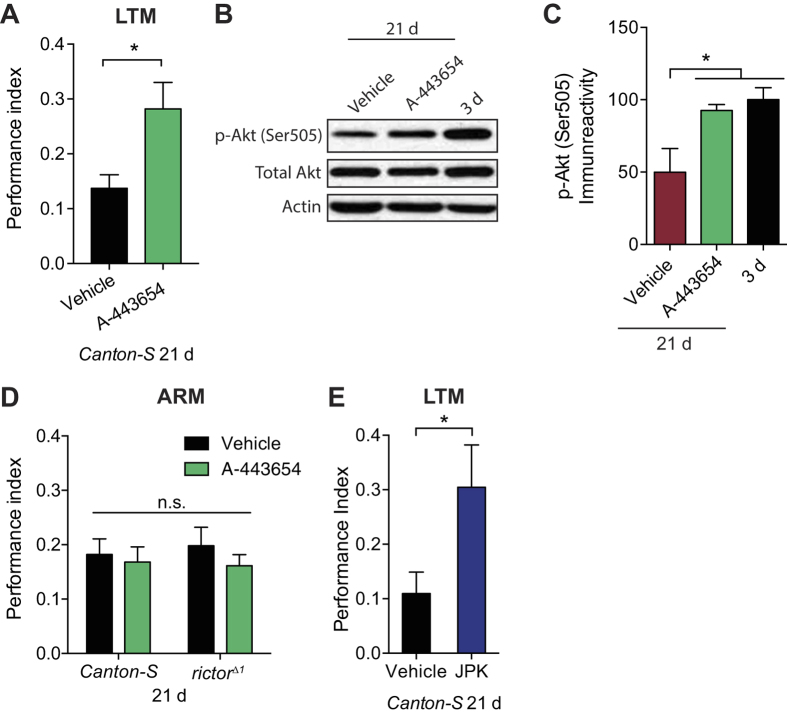
Direct activation of dTORC2 signaling selectively restores LTM in aged flies. (**A**) Impaired LTM in aged (21 d) WT flies was improved by treatment with 10 μM A-443654 (n = 7/group, unpaired *t* test, t = 2.67, *P < 0.05). (**B**) Administration of 10 μM A-443654 significantly enhanced dTORC2 activity (p-Akt Ser505) in aged (21 d) WT flies to comparable levels in young (3 d) WT flies. Actin is the loading control and the blot is representative of three independent replicates. (**C**) Normalized data of p-Akt Ser505 levels (p-Akt Ser505 normalized to total Akt levels; n = 4 per group, one-way ANOVA and between-group comparisons using Bonferroni’s test, F_2,9_ = 6.165, *P < 0.05). (**D**) Administration of 10 μM A-443654 did not have a significant effect on massed training-induced ARM in aged (21 d) WT and *ricto*r^Δ1^ flies (n = 11 per group, two-way ANOVA, F_1,34_ = 0.762, P = 0.389). **(E**) JPK administration improved LTM in aged (21 d) WT flies (n = 6 per group, unpaired *t* test, t = 2.234, *P < 0.05). All data are presented as mean ± s.e.m.

If the reduced dTORC2 activity in the brains of aged flies is responsible for their cognitive decline, then its up-regulation activity should ameliorate these memory deficits. Indeed, administration of a small molecule that activates mTORC2-mediated p-Akt (Ser473) (A-443654)^38^ markedly restored LTM performance in aged flies (Fig. 2A). The improved LTM storage was accompanied by a significant increase in dTORC2 activity levels in aged wild-type flies (Figs. 2B-C), but not in dTORC2-deficient flies (*rictor*^Δ1^; Supplementary Fig. S1A online), as measured by the phosphorylation of Akt at Ser505. Furthermore, A-443654 treatment failed to restore LTM in aged *rictor*^Δ1^ mutants (see Supplementary Fig. S1B online) and ARM in aged WT flies (Fig. 2D), demonstrating that A-443654 specifically restores LTM in aged flies through the activation of dTORC2. Importantly, A-443654 treatment had no effect on sensory acuity, since both vehicle and drug-treated groups showed normal avoidance of the electric shock and the odorants 4-methylcyclohexanol (MCH) and 3-octanol (OCT; see Supplementary Fig. S2 online).

mTORC2 has recently been shown to promote actin-dynamic mediated changes in synaptic strength and LTM formation^15^. Given the reduction in dTORC2 activity in aged flies (Figs. 1A-B), we predicted that actin polymerization should be reduced in aged flies and that jasplakinolide (JPK), a compound that directly promotes actin polymerization^39^, should also restore LTM in these flies. Indeed, similar to treatment with A-443654, administration of JPK restored LTM in aged flies (Fig. 2E). Taken together, reduced dTORC2-mediated actin polymerization in aged flies contributes, at least in part, to the LTM impairment, which can be reversed by increasing either dTORC2 or actin polymerization pharmacologically.

### Direct activation of dTORC2 and actin polymerization enhances LTM in young flies

**Figure 3:**
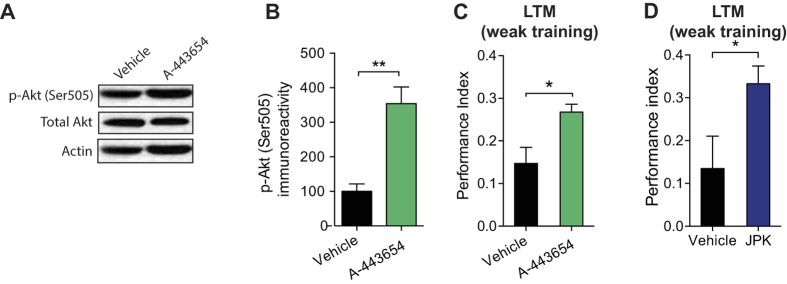
Increasing dTORC2 activity or directly promoting actin polymerization in young flies enhances LTM generated by a weak training protocol. (**A**) Western blots reveal significantly increased dTORC2 activity (p-Akt Ser505) in brain extracts from young (3 d) WT flies treated with 10 μM A-443654. Actin is the loading control and the blot is representative of three independent replicates. (**B**) Normalization of p-Akt Ser505 levels (p-Akt Ser505 normalized to total Akt levels; n = 3 per group, unpaired *t* test, t = 4.815, **P < 0.01). (**C**) LTM is enhanced in young (3 d) WT flies treated with 10 μM A-443654 and given a weak training protocol (1 trial; n = 9 per group, unpaired *t* test, t = 2.758, *P < 0.05). (**D**) JPK enhanced LTM in young (3 d) WT flies given a single training trial (n = 7 per group, unpaired *t* test, t = 5.354, *P < 0.05). All data are presented as mean ± s.e.m.

In young flies with normal levels of dTORC2, direct pharmacological activation of dTORC2 signaling might not further enhance LTM generated by a strong training protocol (five training sessions with a 15-min rest interval between each). Consistent with this prediction, increasing dTORC2 activity *via* A-443654 had no effect on LTM in young flies (see Supplementary Fig. S3 online), suggesting that the effect of A-443654 in flies is age-specific. However, direct activation of dTORC2 signaling in young flies given a weak training protocol, which normally does not elicit LTM, might convert relatively short-lasting memories into long-lasting ones. In young flies, a weak training protocol generates a weak memory (Fig. 3C). As we predicted, young flies treated with A-443654 and the same weak training protocol exhibited increased dTORC2 activity in the brain (Figs. 3A-B) and enhanced LTM (Fig. 3C) compared to vehicle-treated controls. These results indicate that a transient activation of dTORC2 activity promotes LTM formation and counteracts cognitive decline in the adult animal. Finally, promoting actin polymerization with JPK also significantly enhanced LTM in young flies given a weak training protocol (Fig. 3D). These data support the notion that direct activation of dTORC2-mediated actin polymerization enhances LTM formation.

### Direct activation of mTORC2 restores LTM in aged- mice

**Figure 4:**
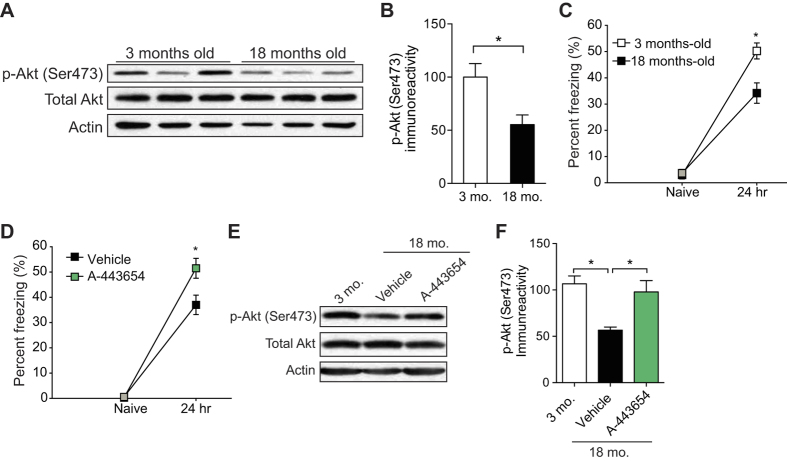
mTORC2 activity decreases significantly with age in mice and activating mTORC2 restores LTM in aged WT mice. (**A**) Western blots show reduced mTORC2 activity (p-Akt Ser473) in the hippocampus of 18-month-old WT mice compared to 3-month-old WT mice. Actin is the loading control and the blot is representative of three independent replicates. (**B**) Normalized mTORC2 activity (p-Akt Ser473 normalized to total Akt levels; n = 3 per group, unpaired *t* test, t = 2.871, *P < 0.05). (**C**) At 24 hr after a strong training protocol [two pairings of a tone (2800 Hz, 85 dB, 30 s) with a co-terminating foot-shock (0.7 mA, 2 s)] contextual fear LTM was impaired in 18-month-old mice, compared to the LTM of young 3-month-old mice (n = 8 per group, two-way repeated measures ANOVA followed by Bonferroni’s test for between-group comparison, F_1,30_ = 8.77, *P < 0.05). (**D**) Compared to vehicle-controls, a single injection of A-443654 (2.5 mg/kg i.p.) immediately after a strong training protocol, enhanced contextual fear LTM in aged mice (n = 9/vehicle, n = 11/A-443654, two-way repeated measures ANOVA followed by Bonferroni’s test for between-group comparison, F_1,20_ = 4.945, *P < 0.05) and (**E**) increased mTORC2 activity to comparable levels in young mice. Actin is the loading control and the blot is representative of three independent replicates. **(F)** Normalized mTORC2 activity (p-Akt Ser473 normalized to total Akt levels; n = 3 per group, one-way ANOVA followed by Bonferroni’s test for between-group comparison, F_2,6_ = 9.107, t = 2.489, *P < 0.05) in the hippocampus. All data are presented as mean ± s.e.m.

Because mTORC2 and its role in memory consolidation are evolutionarily conserved^15^, we wondered whether deficient mTORC2 also contributes to age-related memory impairment in mice. First, we measured the activity of mTORC2 in the hippocampus of young (3 month old) and old (18 month old) mice. Matching our findings in flies, mTORC2 activity—as measured by the levels of phosphorylated Akt at Ser473^30^, was indeed decreased in the hippocampus, a key brain region implicated in LTM formation, of aged mice compared to young mice (Figs. 4A-B).

We next tested LTM in young and aged mice using contextual fear conditioning, a form of Pavlovian learning^40^. In this behavioral assay, a mouse learns to associate an aversive stimulus (US), such as a mild foot shock, with a salient environmental cue (CS), such as the context of the test chamber. The mouse is reintroduced into the test chamber 24 hours after training and fear responses (“freezing”) were recorded. The percentage of time spent freezing was taken as an index of the strength of the association between conditioned and unconditioned stimuli. Young and aged mice showed similar amounts of freezing behavior before training (naïve). However, when examined 24 h after training, contextual fear LTM was significantly impaired in aged mice (Fig. 4C), consistent with previous reports^4^,^41^,^42^,^43^. Remarkably, aged mice injected intraperitoneally with A-443654 immediately after training froze significantly more than vehicle-injected controls, indicating that their contextual LTM was ameliorated (Fig. 4D). Such injections of A-443654 significantly increased mTORC2 activity in the hippocampus of aged mice compared to vehicle-injected controls (Figs. 4E-F). Hence, restoration of TORC2 activity functions in an evolutionarily conserved manner to restore the LTM decline in both flies and mice.

## Discussion

Normal aging of the brain is associated with consistent alterations in cognition^3^,^4^, but the molecular mechanisms underlying age-related changes in the brain are not well understood. Our findings suggest that mTORC2 deficiency plays a crucial role in age-dependent memory impairment in both flies and mice. We found that mTORC2 activity is significantly reduced in the brain of aged flies and mice (Figs. 1A and 4A). Remarkably, acute pharmacological activation of mTORC2 by A-443654 is sufficient to reverse LTM impairments in both model systems (Figs. 2A and 4D). Interestingly, A-443654 increased mTORC2 activity, as determined by p-Akt (Ser473), in aged mice to comparable baseline levels in young mice (Figs. 4E-F). The comparison of freezing scores in Figs. 4C-D indicate that A-443654 fully restores LTM performance in aged mice.

In aged flies, A-443654 increased dTORC2 activity to levels comparable to those in young flies (Figs. 2B-C). While A-443654 restored LTM in aged flies, this did not reach the LTM performance of young flies (Fig. 2A). We speculate that, given that A-443654 is fed to aged flies after a starvation period (and prior to training), starvation of aged flies may prevent a complete rescue of LTM. Alternatively, there could be additional signaling pathways required in aged flies to boost LTM performance. Given that human brain aging is accompanied by memory loss and reduced synaptic connectivity^3^,^4^,^7^,^8^ and the molecular mechanisms underlying memory formation are evolutionarily conserved^34^, deficient mTORC2 activity may account, at least in part, for the age-associated inability to consolidate long-term memories. If so, drugs such as A-443654, that promote mTORC2 activity, could ameliorate some of the cognitive deficits associated with aging.

The small molecule A-443654 was initially discovered to be a potent inhibitor of Akt targets and tumor growth in a number of *in vivo* tumor models^38^,^44^. At the doses required to inhibit tumor growth, significant inhibition of the Akt pathway was observed. Interestingly, A-443654 causes an mTORC2-mediated hyperphosphorylation of Akt at Ser473^38^. Although we cannot rule out this possibility, it is unlikely that A-443654 restores LTM formation due to the abrogation of Akt-mTORC1 pathway given that previous studies have shown that this signaling pathway is crucial for LTM formation^45^,^46^. Indeed, mTORC1 activity was significantly decreased in the hippocampus of aged mice (see Supplementary Fig. S4 online) and A-443654 had no effect on mTORC1 activity^38^. More importantly, in the absence of mTORC2, A-443654 lost the ability to enhance memory (see Supplementary Fig. S1 online)^15^, indicating that the memory-enhancing effects of A-443654 are mTORC2-mediated.

A small subset of key signaling pathways can be genetically manipulated to enhance cognition in normal animals^47^. Our findings show that memory can also be enhanced in both normal and pathological states by targeting proteins involved in synaptic structural changes such as mTORC2 and actin dynamics. Previous evidence indicates that actin polymerization is required for LTM formation and functions to modify neural synaptic transmission and morphology, resulting in long-lasting changes in synaptic efficacy and behavior^24^. We postulate that in response to neuronal activation, an increase in mTORC2-mediated actin polymerization allows for structural rearrangements and the trafficking and insertion of AMPA receptors at the post-synaptic density. Consistent with this idea, actin polymerization and spine density are reduced in mTORC2-deficient mouse hippocampal neurons^15^. In addition, both L-LTP-inducing protocols and behavioral learning transiently increase mTORC2 activity in the hippocampus^15^. Thus, it is possible that brain aging hijacks mTORC2 by reducing its activity. The deficient mTORC2-mediated changes in actin polymerization may not be sufficient to achieve proper trafficking and insertion of AMPA receptors and thus bring about synaptic potentiation and LTM formation. Indeed, a recent study on rhesus macaques revealed an age-related impairment in maintenance of GluA2-containing AMPA receptors in the hippocampus and this is coupled to memory impairment^48^.

It is widely accepted that aging is a major risk factor for neurodegeneration. However, a crucial question is whether age-related changes in mTORC2 activity are also responsible for the pathological processes associated with neurodegenerative disorders such as Alzheimer’s disease. Previous studies have shown that mTORC2 activity is altered in several neurodegenerative disorders including Huntington’s disease^49^, Parkinson’s disease^19^, and Alzheimer’s disease^18^. Further investigation into the mechanism by which mTORC2 regulates age-associated memory impairment may be the key to the understanding the role of mTORC2 in the onset and progression of neurodegenerative disorders in humans.

In conclusion, our work identifies defective mTORC2 signaling as a novel, evolutionarily conserved mechanism of age-associated memory impairment. Although we are only beginning to understand the unique functions of mTORC2 in the brain, therapeutic approaches targeting the mTORC2/Akt signaling pathway may have a profound impact in the prevention and treatment of age-related neurological diseases. Interestingly, a recent study found that depletion of Rictor and mTORC2 activity in adult male mice significantly decreased lifespan^26^. While further investigation is needed, it is possible that transient activation of mTORC2 activity may have a beneficial effect on animal survival. Therefore, treatment with A-443654 to enhance mTORC2 activity, may potentially act to rejuvenate the brain, delay the rate of brain aging and help preserve memory in the old. Compounds similar to A-443654, which have been used in human clinical trials for cancer progression^50^, may prove beneficial for treating a wide array of age-related diseases.

## Materials and Methods

### Mice

The following mouse lines were used: C57BL/6 young (3 month) mice and C57BL/6 aged (18 month) mice (National Institutes of Aging). The mice were kept on a 12-h light/dark cycle, and the behavioral tests were always conducted during the light phase of the cycle. The mice had access to food and water *ad libitum*, except during tests. This study was conducted in strict accordance to the recommendations in the Guide for the Care and Use of Laboratory Animals put forth by the National Institutes of Health. Animal care and experimental procedures were approved by the animal care committee of Baylor College of Medicine, according to US National Institutes of Health Guidelines.

### *Drosophila* husbandry

Flies were reared on a standard medium at 25 °C, 60% relative humidity, and a 12 hr light/dark cycle. Wild-type *Canton-S* and *rictor*^Δ1^ mutants^51^ were used for this study. The *rictor*^Δ1^ mutants were out-crossed into a wild-type *Canton-S* background before behavioral experimentation. For behavioral studies, 50 flies of mixed sex were aliquoted into food vials and transferred to fresh food vials every 3 or 4 d until they reached the age for training and testing.

### Behavior

#### Contextual fear conditioning

Fear conditioning was performed as previously described^40^,^52^. Mice were first handled for 5 min for 3 d and then habituated to the conditioning chamber for 20 min for another day. On the training day, after 2 min in the conditioning chamber, mice received two pairings of a tone (2,800 Hz, 85 dB, 30 s) with a co-terminating foot shock (0.7 mA, 2 s), after which they remained in the chamber for an additional minute and were then returned to their home cages. At 24 h after training, mice were tested for freezing (immobility except for respiration) in response to the training context (training chamber). Freezing behavior was hand-scored at 5-s intervals by an observer blind to the genotype. The percentage of time spent freezing was taken as an index of learning and memory. For A-443654 administration, A-443654 was initially dissolved in DMSO to make a 40 mM stock. The compound was then freshly diluted in saline and injected intraperitoneally at a dose of 2.5 mg per kg immediately after training.

#### Drosophila aversive olfactory conditioning

The negatively reinforced olfactory learning procedure was carried out according to established protocols, with some modifications^36^,^53^. Briefly, the training odorants were 0.2% octanol (vol/vol, Sigma-Aldrich) and 0.12% methylcyclohexanol (vol/vol, Sigma-Aldrich) diluted in mineral oil. For each training trial, flies were exposed to the first odorant (CS^+^) and twelve 1.25-s pulses of electric shock at 90 V for 1 min. This was followed by a 1-min presentation of the second odorant, which was not paired with shocks (CS^−^). Both spaced and massed training schedules had five training trials. The inter-trial interval was 15 min for spaced training and 30 s for massed training. Memory was then tested in a T maze 24 h after the final training trial and the performance index calculated as: (number of flies that chose the CS^−^–the number of flies that chose the CS^+^)/(total number of flies in both tubes), as previously described^53^. Octanol and methylcyclohexanol alternated as the CS^+^, and the results were averaged.

For A-443654 and JPK administration, flies were starved for eight hours prior to training and then transferred to vials containing vehicle (1% DMSO +5% sucrose) or 10 μM A-443654/50 nM JPK +5% sucrose for 1 hr. Flies were then transferred to standard medium for 0.5 hr and then subjected to training. For sensory controls, groups of flies were given 2 min to choose between the odorant and air or shocked and non-shocked side in a T-maze. The odorant concentrations and shock voltages were the same as in the LTM experiments. Avoidance was calculated as: (number of flies that chose air/non-shocked side—the number of flies that chose the odorant/shocked side)/(total number of flies in both tubes).

### Western Blotting

Samples were homogenized in buffer containing 200 mM HEPES, 50 mM NaCl, 10% glycerol (vol/vol), 1% Triton X-100 (vol/vol), 1 mM EDTA, 50 mM NaF, 2 mM Na_3_VO_4_, 25 mM β-glycerophosphate and EDTA-free complete ULTRA tablets (Roche). A total of 30 μg of protein per sample was resolved on SDS-PAGE (10%), transferred onto nitrocellulose membranes and blotted as previously described^15^. Western blots were quantified by densitometry using the ImageJ software.

### Antibodies

For primary antibodies, we used antibodies to p-S6 ribosomal protein (Ser240/244, #4856, 1:1000), p-Akt (Ser473, #9271, 1:1,000), total S6 ribosomal protein (#2217, 1:1,000), total Akt (#9272, 1:1,000), β-actin (#3700, 1:1,000), and *Drosophila* p-Akt (Ser505, #4504, 1:1,000) (all from Cell Signaling Technologies).

### Statistical analysis

All data are presented as means ± s.e.m. The sample size used to result in statistically significant differences was calculated using standard power calculations with α = 0.05 and a power of 0.8. Statistics were calculated by means of Prism (GraphPad software). Shapiro-Wilk normality test was used to test for normal distribution and all means were compared with Student’s *t* test, one-way or two-way ANOVA and corrected for multiple comparisons (Bonferroni). *P* < 0.05 was considered significant.

## Additional Information

**How to cite this article**: Johnson, J. L. *et al.* TORC2: a novel target for treating age-associated memory impairment. *Sci. Rep.* **5**, 15193; doi: 10.1038/srep15193 (2015).

## Supplementary Material

Supplementary Figures
