# Supplementary material for: TORC2: a novel target for treating age-associated memory impairment
Source: Sci Rep. 2015 Oct 22;5:15193. doi: 10.1038/srep15193 (PMC4614817; doi:10.1038/srep15193)
Supplement: Supplementary Figures [file srep15193-s1.pdf]

TORC2: a novel target for treating age-associated memory impairment

Jennifer L. Johnson[1,2], Wei Huang[1,2], Gregg Roman[3,4], Mauro Costa-Mattioli[1,2]*

[1]Department of Neuroscience, [2]Memory and Brain Research Center (MBRC), Baylor College of Medicine, Houston, TX 77030, USA.

[3]Biology and Biochemistry Department, [4]Biology of Behavior Institute, University of Houston, Houston, TX 77004, USA.

*To whom correspondence should be addressed: costamat@bcm.edu

Supplementary Materials

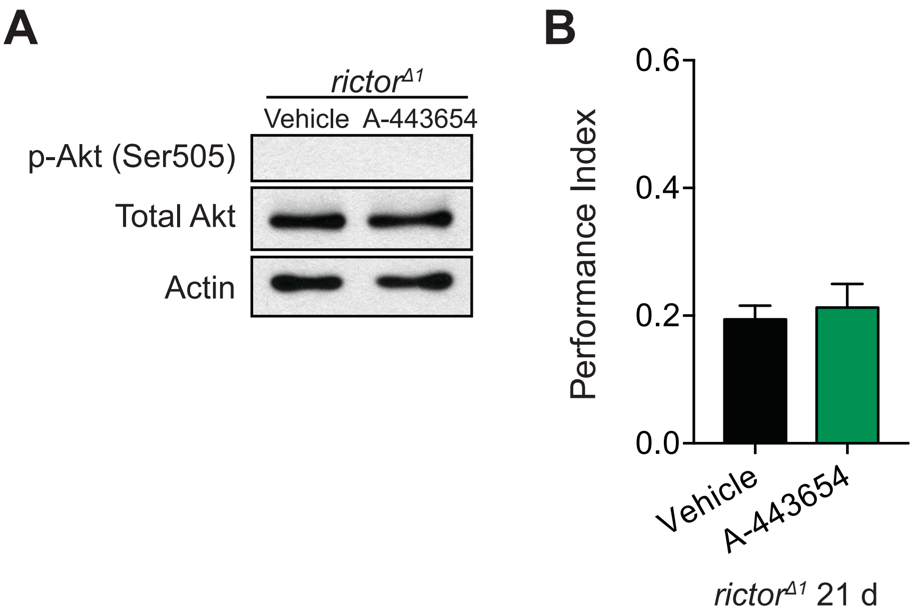

Supplementary Figure 1. Direct activation of dTORC2 by A-443654 does not affect LTM in aged flies lacking dTORC2. (A) Administration of 10 μM A-443654 had no effect on dTORC2 activity (p-Akt Ser505) in *rictor*$^{\Delta 1}$ flies. Actin is the loading control and the blot is representative of three independent replicates. **(B)** Administration of 10 μM A-443654 did not have a significant effect on LTM in aged *rictor*$^{\Delta 1}$ flies (n=6-9 per group, unpaired *t* test, t= 0.436, P= 0.673).

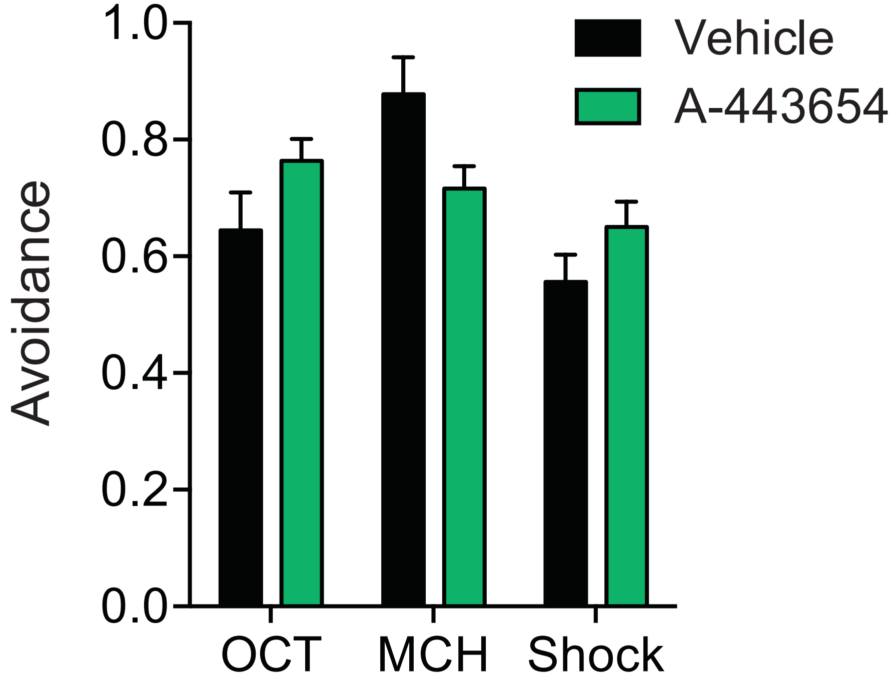

Supplementary Figure 2. Direct activation of dTORC2 by A-443654 does not affect sensory acuity in aged flies. Vehicle-treated controls and flies treated with 10 µM A-443654 exhibited normal avoidance of electric shock (90 V dc; n=14 per group, t=1.581, P=0.145) or the odorants 4-methylcyclohexanol (0.12% MCH; n=6 per group, t= 2.178, P=0.06) and 3-octanol (0.2% OCT; n=6 per group, t=1.487, P=0.15). In electric shock-avoidance controls, one arm of the T-maze was electrified with 90 V dc for 2 min. Avoidance was calculated as described in Methods. Data are mean ± s.e.m.

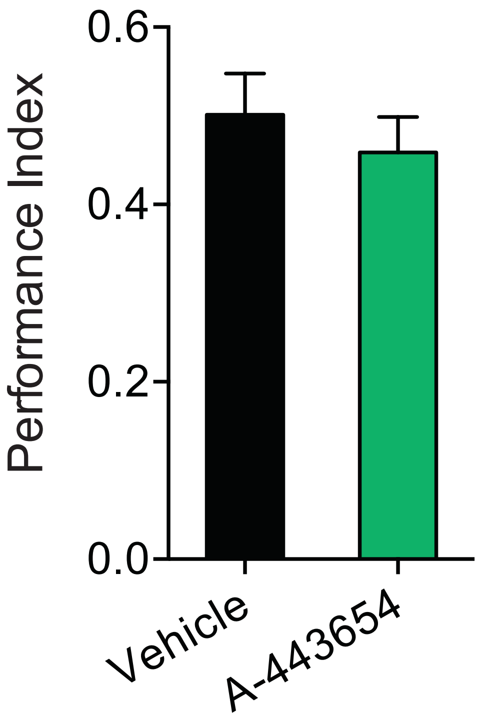

Supplementary Figure 3. A-443654 has no effect on LTM in young flies. Spaced training induced similar LTM in young flies treated with vehicle or 10 μM A-443654 (n= 7 per group, t= 0.698, P= 0.499). Data are mean ± s.e.m.

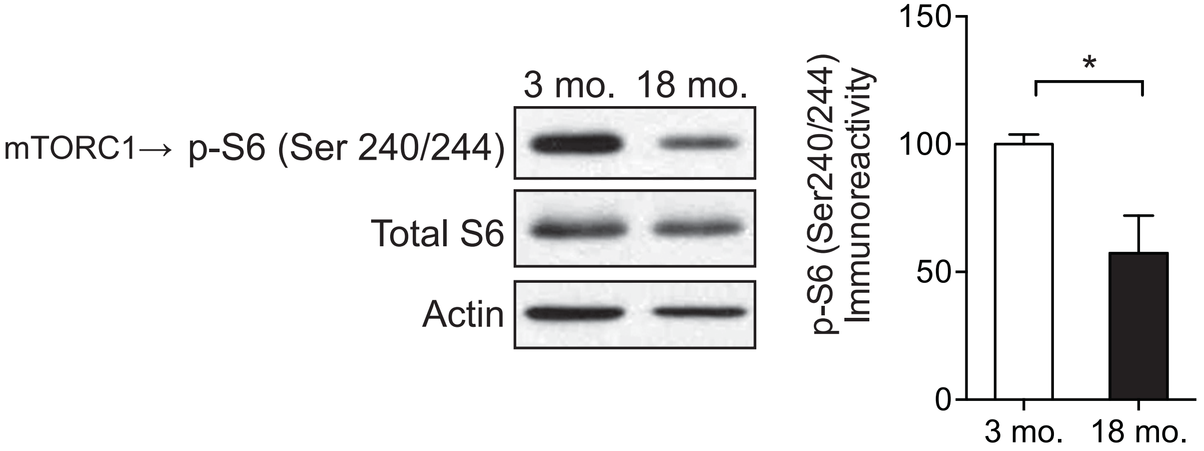

Supplementary Figure 4. mTORC1 activity decreases significantly with age in mice. Western blots show significantly reduced levels of mTORC1 activity (p-S6 Ser240/244) in the hippocampus of 18-month-old WT mice compared to 3-month-old WT mice (n= 3 per group, t= 2.797, *P<0.05). Data are mean ± s.e.m.
